# A Recombinant Rift Valley Fever Virus Glycoprotein Subunit Vaccine Confers Full Protection against Rift Valley Fever Challenge in Sheep

**DOI:** 10.1038/srep27719

**Published:** 2016-06-14

**Authors:** Bonto Faburay, William C. Wilson, Natasha N. Gaudreault, A. Sally Davis, Vinay Shivanna, Bhupinder Bawa, Sun Young Sunwoo, Wenjun Ma, Barbara S. Drolet, Igor Morozov, D. Scott McVey, Juergen A. Richt

**Affiliations:** 1 Department of Diagnostic Medicine and Pathobiology, College of Veterinary Medicine, Kansas State University, Manhattan, Kansas, USA; 2 United States Department of Agriculture, Agricultural Research Service, Arthropod Borne Animal Disease Research Unit, Manhattan, Kansas, USA.

## Abstract

Rift Valley fever virus (RVFV) is a mosquito-borne zoonotic pathogen causing disease outbreaks in Africa and the Arabian Peninsula. The virus has great potential for transboundary spread due to the presence of competent vectors in non-endemic areas. There is currently no fully licensed vaccine suitable for use in livestock or humans outside endemic areas. Here we report the evaluation of the efficacy of a recombinant subunit vaccine based on the RVFV Gn and Gc glycoproteins. In a previous study, the vaccine elicited strong virus neutralizing antibody responses in sheep and was DIVA (**d**ifferentiating naturally **i**nfected from **v**accinated **a**nimals) compatible. In the current efficacy study, a group of sheep (*n* = 5) was vaccinated subcutaneously with the glycoprotein-based subunit vaccine candidate and then subjected to heterologous challenge with the virulent Kenya-128B-15 RVFV strain. The vaccine elicited high virus neutralizing antibody titers and conferred complete protection in all vaccinated sheep, as evidenced by prevention of viremia, fever and absence of RVFV-associated histopathological lesions. We conclude that the subunit vaccine platform represents a promising strategy for the prevention and control of RVFV infections in susceptible hosts.

Rift Valley fever virus (RVFV), a member of the *Phlebovirus* genus in the family *Bunyaviridae*, is a mosquito-borne zoonotic pathogen that causes recurrent outbreaks in ruminants and humans in Africa and the Arabian Peninsula^1^. It is classified as an overlap select agent and risk group-3 pathogen by the Centers for Disease Control and Prevention and the United States Department of Agriculture. Like all members of the family *Bunyaviridae*, RVFV has a tripartite single-stranded negative RNA genome composed of small (S), medium (M) and large (L) segments. The S segment encodes the nucleocapsid protein (N) and the non-structural protein NSs. The M segment encodes the two glycoproteins, Gn and Gc, the 78-kDa protein and the non-structural protein, NSm. The L-segment encodes the RNA-dependent RNA polymerase^2^.

The presence of competent vectors in non-endemic areas presents significant risk of introduction and further spread of RVFV^3^,^4^,^5^. Although RVFV has the potential to cause severe epizootics in livestock in North America or Europe, there is currently no fully licensed safe veterinary vaccine available to control the disease in the event of potential virus introduction or prevent a disease outbreak. In endemic regions in Africa and the Arabian Peninsula, RVF in livestock has been controlled primarily by using the live-attenuated Smithburn strain or inactivated whole virus^6^. The Smithburn vaccine is highly immunogenic but is teratogenic in pregnant sheep and cattle^7^,^8^. Whole-virus formalin inactivated vaccines are generally less immunogenic^9^,^10^ and their production is associated with risk of human exposure to wild-type virulent virus. A natural attenuated isolate, Clone 13, from a benign RVF case in the Central African Republic^11^, and a chemically attenuated virus, MP12, derived from ZH548, an Egyptian wild-type isolate^12^, have been evaluated for efficacy^13^,^14^,^15^. Although these attenuated vaccines have shown promising results^16^, safety issues associated with their use in non-endemic regions remain a major concern^17^. Another drawback of the live-attenuated RVFV vaccines is that they do not allow for differentiation of infected from vaccinated animals (DIVA). DIVA-compatibility is critical if a vaccination strategy is to be used to support efforts to control and eradicate wild-type RVFV in non-endemic areas.

In contrast to the live vaccines, no major safety issues are associated with the use of subunit vaccines. Efforts to develop such vaccines include recombinant baculovirus expressed protein-based vaccines^18^,^19^,^20^, DNA vaccines^21^,^22^, virus-like particles^20^,^23^,^24^,^25^, virus replicon particles^26^,^27^,^28^ and virus-vectored vaccines^24^,^29^,^30^,^31^,^32^. However, only a few of these vaccine candidates have been evaluated for efficacy in natural host species, for example sheep^24^,^28^,^32^, as reviewed in Lorenzo *et al*.^33^.

In a previous study, we described induction of RVFV neutralizing antibody responses using the recombinant RVFV Gn/Gc subunit vaccine in an immunogenicity trial in sheep^19^. This indicates that the surface glycoproteins, Gn and Gc, carry epitopes that elicit production of neutralizing antibodies, the only established correlates of protective immunity against RVFV infection^19^,^34^,^35^,^36^. Furthermore, in a recent study, we demonstrated the suitability of sheep (*Dorper x Katahdin cross*) as an experimental model for RVF using a wild type strain (Kenya 2006-128b-15, Ken06) as challenge inoculum^37^. The current work is a logical continuation of this work aimed at evaluating the efficacy of the recombinant Gn/Gc subunit vaccine candidate to protect against heterologous virus challenge in sheep.

## Results

### Clinical evaluations

**Figure 1:**
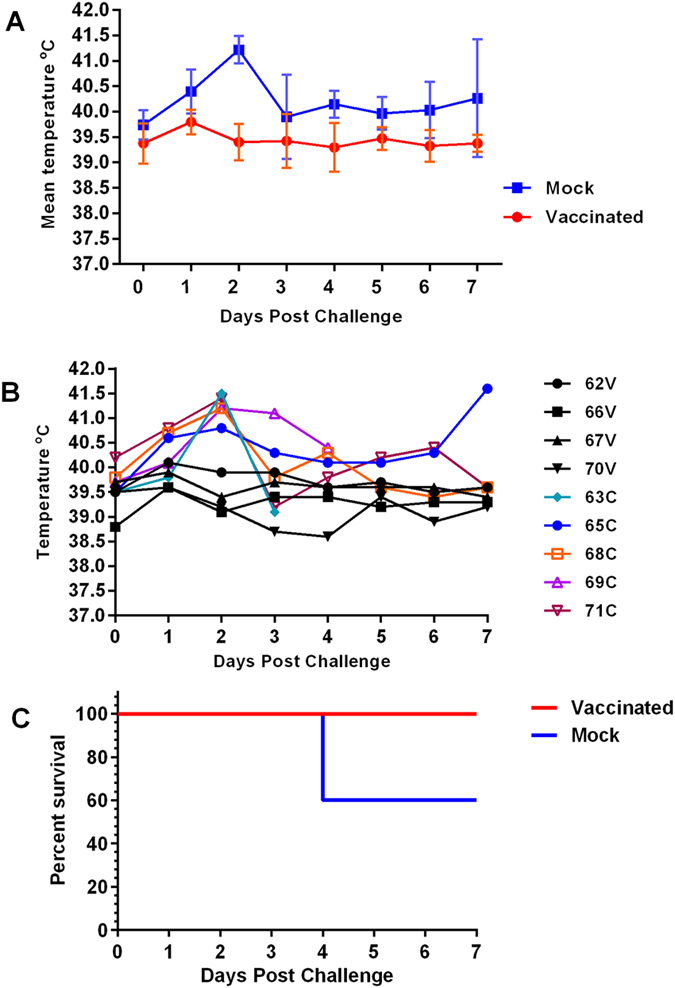
Temperature responses in vaccinated and mock-vaccinated sheep following virulent RVFV challenge. (**A**) Shows mean rectal temperature responses in vaccinated and mock-vaccinated (mock) groups; mean rectal temperatures of mock-vaccinated group were significantly higher than vaccinated group (*P* = 0.00024). (**B**) Shows rectal temperature responses in individual animals following virulent RVFV challenge; V = vaccinated; C = mock-vaccinated. (**C**) Survival analysis of sheep challenged with wild type RVFV.

Two groups of sheep (*n* = 5 per group), Dorper x Katahdin cross, aged 4–5 months, were used in the study. Group 1 was vaccinated subcutaneously with 2 ml of the vaccine and group 2 received placebo (mock-vaccinated) as described in the Methods. Two out of 10 animals showed increases in rectal temperatures following vaccination. The two animals, #64 (vaccinated) and #63 (mock-vaccinated) developed transient increased temperatures of 40.6 °C and 41 °C, respectively, on 2 days post-vaccination (dpv). Since one animal was vaccinated and the other, mock-vaccinated, this increase in temperature was most likely not due to the RVFV subunit vaccine. Their temperatures normalized the following day (on 3 dpv). A mild localized swelling was detected at the vaccination site in both vaccinated and mock-vaccinated sheep. This quickly resolved and was most likely associated with vaccine/adjuvant depot. No erythema or abscesses were detected throughout the 35-day observation period. Following virus challenge, animals in the control (mock-vaccinated) group responded with pyrexia and maintained significantly higher mean rectal temperatures than the vaccinated group throughout the challenge study (*P* < 0.001) (Fig. 1A). Peak temperatures occurred in all unvaccinated sheep at 2 days post-challenge (dpc) followed by a decline (Fig. 1A,B). Two control sheep, #63 and #69, died of acute RVF at 4 dpc indicating a survival rate of 60% (Fig. 1C). Both sheep had significantly higher rectal temperatures, 41.5 °C and 41.2 °C, respectively, at 2 dpc compared to their baseline values at 0 dpc (*P* < 0.05) (Fig.1B). In contrast, none of the sheep in the vaccinated group showed clinical symptoms of virulent RVFV infection.

### Blood chemistry

**Figure 2:**
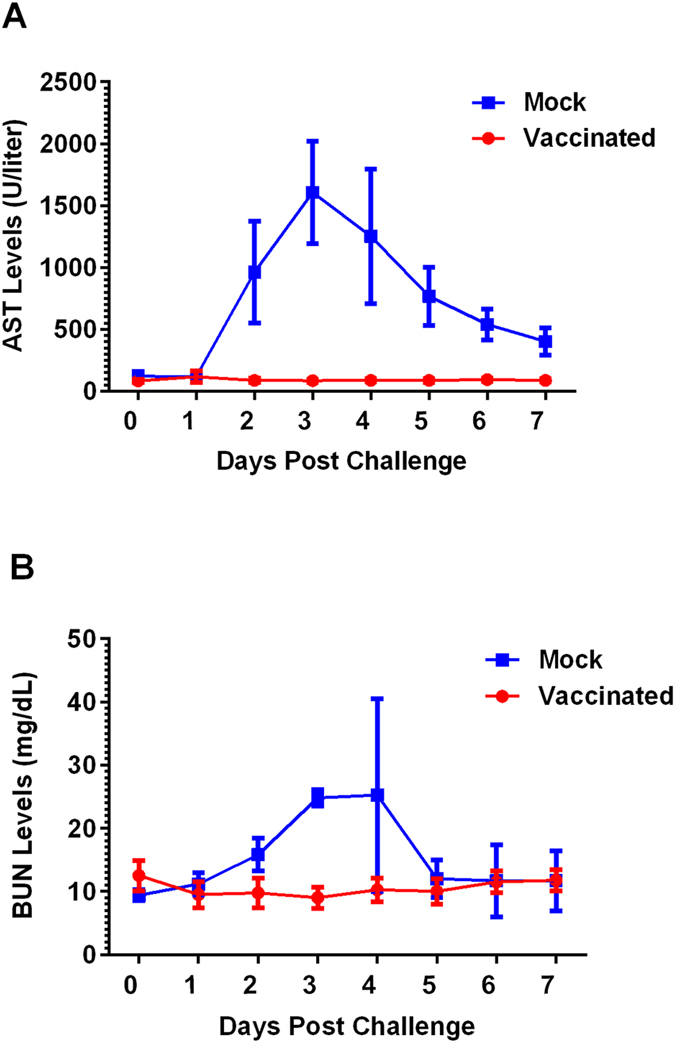
Blood chemistry analysis of serum samples from vaccinated and mock-vaccinated sheep following virulent RVFV challenge. (**A**) Illustrates mean concentrations of serum AST; the mock group responded with significantly higher serum AST concentrations (*P* < 0.0001). (**B**) Illustrates mean concentrations of BUN in response to virulent challenge. The mock group responded with significantly higher serum BUN concentrations (*P* = 0.0001).

Blood biochemistry changes in response to viral challenge are presented in Fig. 2A,B. Sheep in the mock-vaccinated group responded with significantly higher concentrations of AST post-challenge compared to the vaccinated group (*P* = 0.0167) (normal range = 80–280 U/liter). Notably, the mock-vaccinated group demonstrated sharp increases in AST concentrations at 2 dpc with peak levels occurring at 3 dpc. This was followed by a steady decline to near baseline levels from 4 to 7 dpc (Fig. 2A). In contrast, vaccinated animals did not show changes in their serum AST, with their concentrations maintained at baseline levels throughout the study (Fig. 2A). In mock-vaccinated animals, mean BUN concentrations were significantly higher (*P* = 0.02) than in the vaccinated sheep (Fig. 2B) (normal range = 8–20 mg/dL). An increase in BUN concentration was observed at 2 dpc, reaching peak concentration at 3 and 4 dpc; this was followed by a decline at 5 dpc to near baseline levels maintained until the study endpoint, 7 dpc (Fig. 2B). In contrast, BUN concentration values of vaccinated animals remained more or less unchanged with concentrations staying within baseline ranges throughout the post-challenge monitoring period (Fig. 2B).

### Viremia

**Figure 3:**
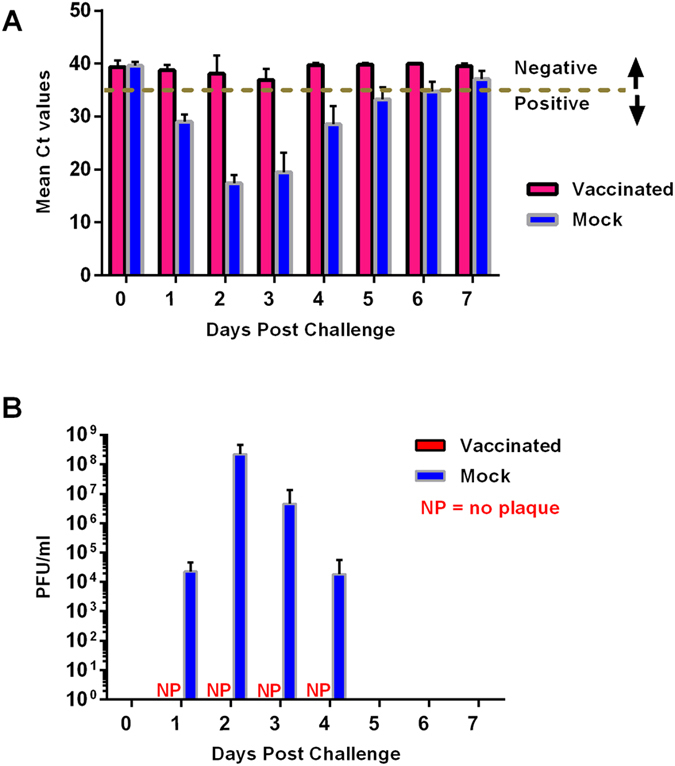
Viremia in vaccinated and mock-vaccinated sheep following virulent RVFV challenge. (**A**) Shows mean QRTPCR Ct values for vaccinated and mock-vaccinated groups. (**B**) Shows quantitation of viremia by plaque assay. Dash line (---) denotes cut-off point. NP = no plaque was isolated. Blanks on the graph (from 5 to 7 dpc) depict no virus was isolated from sheep in either group, vaccinated or mock-vaccinated, by plaque assay.

Viremia was assessed by detecting viral nucleic acid in serum (RNAemia) using qRT-PCR and by virus isolation using plaque assay. Viral nucleic acid was detectable in the control (mock-vaccinated) sheep as early as 1 dpc and remained detectable until 6 dpc. In the mock-vaccinated group, peak RNAemia occurred at 2–3 dpc manifested by correspondingly low Ct values (Fig. 3A). In contrast, mean Ct values of the vaccinated group were above the Ct cutoff throughout the 7-day monitoring period (Fig. 3A). However, one vaccinated sheep, #71, had a mean Ct value of 34.2, just below the threshold cut-off value of 35, at 3 dpc. Following virulent Ken06 challenge at 0 dpc, all mock-vaccinated sheep developed viremia (mean pfu/ml titer = 2.26 × 10^4^) as determined by virus isolation at 1 dpc. Peak viremia occurred at 2 dpc (mean pfu/ml titer = 2.26 × 10^8^), and viremia remained relatively high at 3 dpc (mean pfu/ml titer = 4.59 × 10^6^) (Fig. 3B). A decline in viremia occurred at 4 dpc (mean pfu/ml titer = 1.85 × 10^4^) followed by clearance of the virus (absence of viremia) in all animals from 5 to 7 dpc (Fig. 3B). In contrast, no virus was isolated at any time point from the serum of any of the vaccinated sheep (Fig. 3B).

### Serological responses

**Figure 4:**
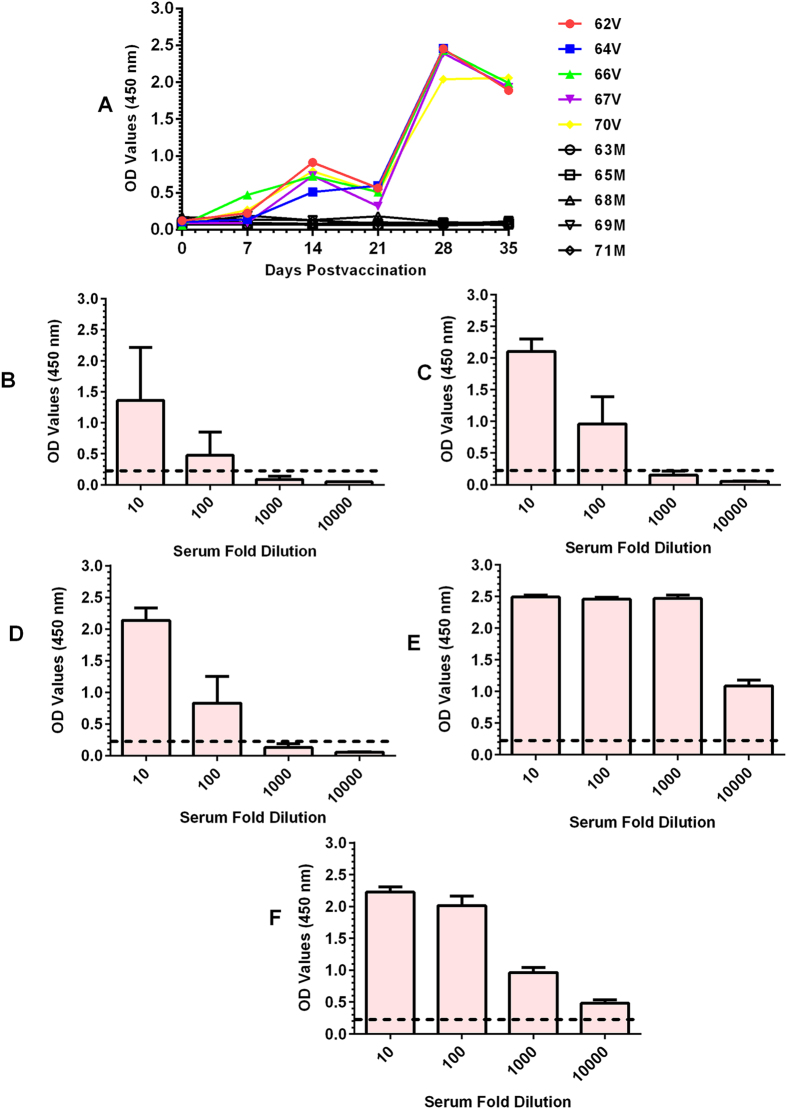
Vaccine-induced immunoglobulin G (IgG) host antibody response demonstrated by antigen-specific (Gn) indirect enzyme-linked immunosorbent assay (ELISA). (**A**) Shows kinetics of Gn-specific IgG antibody response in vaccinated (V) mock-vaccinated (**C**) sheep. The cut-off value for individual sheep: Vaccinated sheep (#62 = 0.266; #64 = 0.116; #66 = 0.083; #67 = 0.101; #70 = 0.082); Mock sheep (#63 = 0.085; #65 = 0.106; #68 = 0.286; #69 = 0.092; #71 = 0.180). Only vaccinated sheep exhibited seroconversion in response to the vaccination. Panel below shows endpoint titers for Gn-ELISA for each time point post vaccination; (**B**) = endpoint titer for 7 dpv; (**C**) = endpoint titer for 14 dpv; (**D**) = endpoint titer for 21 dpv; (**E**) = endpoint titer for 28 dpv; (**F**) = endpoint titer for 35 dpv; (-----) indicates the cut-off value (OD = 0.209).

Indications of vaccine-induced seroconversion were monitored by assessment of the kinetics of Gn-specific IgG antibody responses (Fig. 4A) and endpoint titers for each time point (7, 14, 21, 28 and 35 dpv). All vaccinated animals seroconverted by 14 dpv (Fig. 4A). Following administration of the booster dose at 21 dpv, antibody reactivity increased sharply at 28 dpv. These animals remained strongly seropositive at 35 dpv, when animals were subjected to virulent RVFV challenge (0 dpc). In contrast, none of the mock-vaccinated animals seroconverted during the vaccination period (Fig. 4A). Endpoint titration of Gn-specific antibody responses exhibited time-dependent increase in antibody titers from 7 to 35 dpv, with peak titers occurring at 28 dpv (Fig.4B–F). Positive antibody titers were detected in up to 10,000-fold serum dilutions in 28 and 35 dpv sera, although antibody reactivity in 28 dpv sera yielded higher OD values (Fig. 4E,F).

Development of RVFV neutralizing antibody titers was assessed weekly from 0 to 28 dpv, and then on 0 to 7 dpc. This data is presented as reciprocal values in Table 1. Neutralizing antibody titers (mean titer = 20; *n* = 2) in vaccinated animals were first detected at 7 dpv in two vaccinated sheep (#64 and #66). All vaccinated animals developed neutralizing antibody titers (mean titer = 38; *n* = 5) by 14 dpv. This was followed by a slight increase at 21 dpv (mean titer = 68; *n* = 5). The second vaccination regimen administered at 21 dpv resulted in an anamnestic response in all vaccinated sheep with neutralizing antibody titers of up to ≥1280 mean titer; *n* = 5 (Table 1). In contrast, all mock-vaccinated sheep tested negative for neutralizing antibodies throughout the vaccination period (0–35 dpv or 0 dpc) (Table 1). None of the vaccinated animals displayed increases in their neutralizing antibody titers following virulent challenge at 0 dpc. In contrast, the three surviving mock-vaccinated sheep developed neutralizing antibody titers (mean titer = 133) at 7 dpc (Table 1). One vaccinated sheep, #64, sustained a severe leg injury and was humanely euthanized on 35 dpv.

### Pathology

Mock-vaccinated control animals, #63 and #69, both necropsied at 4 dpc due to sudden death, had disseminated multifocal tan foci (necrosis) and petechiae throughout their hepatic parenchyma. Mock-vaccinated animals, #65, #68 and #71, euthanized at 7 dpc had diffusely pale livers and congested spleens. Additionally, petechiae were visible throughout the hepatic parenchyma of #68 and #71.

**Figure 5:**
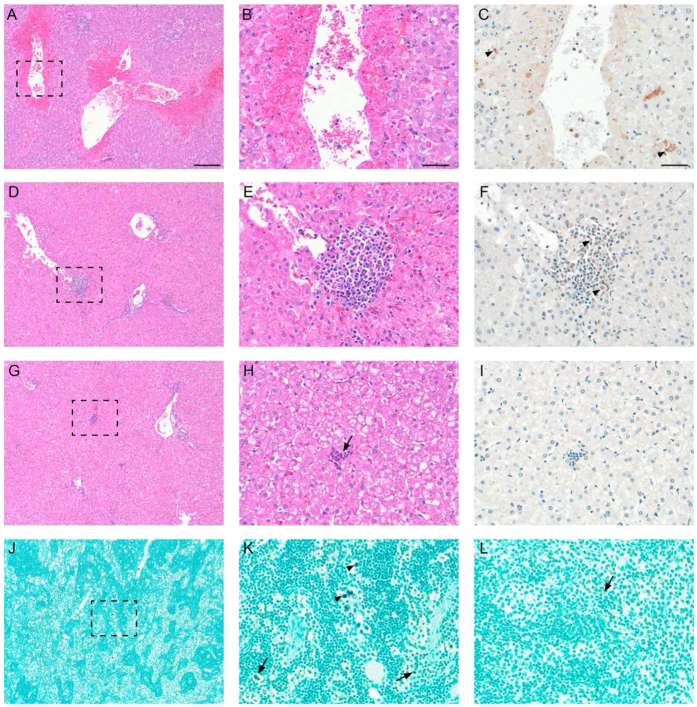
Liver and mesenteric lymph node histopathology and immunohistochemistry. Rift Valley fever virus caused multifocal mid-zonal to central hepatic necrosis accompanied by neutrophilic and histiocytic inflammation. Additionally, hemorrhage was common in the larger necrosis lesions as seen in the 4 dpc mock-vaccinated, virus only study animals. (**A,B**) low and high power fields from a hematoxylin and eosin (H&E) stained section of liver parenchyma from a 4 dpc mock-vaccinated, virus only, sheep, #63, which had severe multifocal necrosis accompanied by hemorrhage involving ~15% of its hepatic parenchyma, hepatic histopathology score of 3. Each broken line box outlines the region shown at higher magnification in the next image. (**C**) Rift Valley fever virus antigen IHC on serial section of same tissue at high power, black arrowheads denote positive labeling for RVFV antigen, red-brown cytoplasmic signal in hepatocytes, inflammatory cells and cellular debris. (**D,E**) H&E stained liver section from a 7dpc mock-vaccinated sheep, #71, which had multifocal, 1–2 mm areas of necrosis with a more lymphohistiocytic infiltrate than #63’s liver and less than 5% of the parenchyma involved, hepatic histopathology score of 2. (**F**). RVFV labeling denoted by black arrowheads. (**G,H**) H&E stained liver section from a 7 dpc vaccinated sheep, #62, black arrow denotes an example of the small neutrophilic inflammatory foci seen in many study sheep that were negative on RVFV IHC (**I**). (**J,K**) Low and high power of RVFV IHC on #63, four dpc mock-vaccinated sheep’s mesenteric lymph node. In order to separate RVFV antigen labeling from endogenous brown pigments, VIP (purple) chromogen was used for RVFV detection and the counterstain was methyl green. Black arrowheads denote RVFV positive cells and black arrows denote apple green colored hemosiderin laden macrophages. (**L**) High power RVFV IHC on #70, seven dpc vaccinated sheep’s mesenteric lymph node that was negative for RVFV antigen. Bar columns 1 and 2 are 200 μm and bar column 3 is 50 μm.

Histopathology and IHC for RVFV antigen findings are summarized in Table 2. The semi-quantitative hepatic histopathology scoring system developed for our prior sheep challenge model studies was applied^37^. We saw three consistent patterns of hepatic histopathologic lesion distribution that correlated with mock-vaccinated animals necropsied at 4 dpc, mock-vaccinated animals necropsied at 7 dpc and vaccinated animals also necropsied at 7 dpc respectively. Mock-vaccinated animals necropsied at 4 dpc had severe liver changes, predominantly multifocal necrosis with minimal inflammation beyond degenerate neutrophils within the necrotic foci (Fig. 5A,B). These lesions were strongly positive for viral antigen (Fig. 5C). The 7 dpc mock-vaccinated animals universally exhibited milder, multifocal hepatic lesions, predominantly foci of lymphohistiocytic inflammation with scattered positive cytoplasmic signals for viral antigen in intralesional macrophages and hepatocytes (Fig. 5D–F). Additionally, some sheep, regardless of treatment group assignment had a milder multifocal liver pathology, small (up to 25 cells) inflammatory foci with a central to midzonal distribution. These foci were predominantly neutrophilic, lacked morphologic evidence of hepatocyte death (Fig. 5G,H) and RVFV antigen IHC on these tissues was convincingly negative (Fig. 5I). Therefore, we didn’t attribute these lesions to RVFV; rather they were consistent with small foci of an unrelated bacterial infection. Also, as reported prior^37^, RVFV attributed hepatic histopathologic changes were present on a background of low numbers of peri-portal lymphoplasmacytic inflammation present in all study animals including uninoculated controls (data not shown).

Both 4 dpc animals (#63 and #69) had splenic lymphoid follicular depletion and perifollicular red pulp necrosis that was positive for viral antigen as well as lymphoplasmacytic interstitial nephritis, evidence of glomeruli filtration of viral antigen and, in the case of animal #63, multifocal tubular necrosis attributable to RVFV. Viral antigen positive cells, macrophages or perhaps dendritic cells were seen in circulation in the lungs and in medullary sinuses of the mesenteric lymph nodes (Fig. 5J,K). Additionally, both these sheep had lung changes, multifocal to coalescing pulmonary edema, including some thickening of alveolar septae with inflammatory cells and alveoli containing plump alveolar macrophages and fibrin. These two sheep’s lymph nodes contained scattered hemosiderin laden macrophages in their medullary sinusoids and sheep #63’s lymph node had scattered sinusoidal histiocytes that were positive for viral antigen (Fig. 5J,K). The three 7 dpc mock-vaccinated sheep’s spleens were histologically within normal limits but scattered perifollicular cells in some of these were positive for viral antigen. Similar to their earlier time-point peers the sinusoids of their mesenteric lymph nodes contained scattered hemosiderin laden macrophages. However, none of these lymphoid tissues were positive for RVFV antigen by IHC. Their lungs were within normal limits and negative for viral antigen. Kidneys were variably involved with mild virus-attributable lesions none of which were RVFV antigen positive. Histopathologic examination of adrenals was all within normal limits for all time-point control animals, except 7 dpc sheep #71 that had a single focus of lymphoplasmacytic inflammation and scattered adrenocortical cell apoptosis, which included rare scattered viral antigen positive cells.

Vaccinated animals, #67 and #70, both had mild lymphoplasmacytic renal pelvic inflammation; both were negative for viral antigen and likely this was an unrelated background lesion, e.g. prior urinary tract insult. Animal #70 additionally had rare scattered pyknotic cellular debris in the center of its splenic lymphoid follicles but the splenic architecture was otherwise undisrupted. No evidence of RVFV antigen was found in lymph nodes (Fig. 5L), spleen, kidney, lung, or adrenal from vaccinated sheep; while all these tissues, except eyes, were variably positive across control sheep as described. No histopathologic lesions were appreciated in the eyes of any of the study’s sheep.

## Discussion

The occurrence of RVFV outbreaks outside the African continent in 2000 in the Arabian Peninsula demonstrated the potential for the virus to spread to non-endemic areas. Unfortunately, there are currently no fully licensed safe and efficacious vaccines for human or livestock use in non-endemic areas. To address this concern, we developed and evaluated the efficacy of a recombinant subunit vaccine candidate composed of RVFV surface glycoproteins, Gn and Gc, adjuvanted with montanide ISA25 VG, in a ruminant model. To date, several vaccine candidates have been developed but few have been tested in target animal models. These include the live vaccines, Clone 13 (licensed for use in South Africa), a natural attenuated RVFV isolate^11^, MP12, chemically attenuated virus derived from RVFV strain ZH548^12^,^38^, NSm/NSs deletion mutants^39^, a modified NSs recombinant MP12^40^,^41^ and an adenovirus- vectored vaccine^32^. There is also safety concern associated with the use of live vaccines in endemic and non-endemic areas due to the inherent risk of potential reassortment with unknown Phleboviruses as well as endemic RVFV strains circulating in resident ruminant livestock populations or the potential to revert to virulence. Subunit vaccines, on the other hand, due to their safety profile, are considered an appropriate vaccine platform for the US and other non-endemic regions to protect domestic livestock against Rift Valley fever. The subunit vaccine evaluated in this study has been shown to be DIVA-compatible^19^; it has shown high efficacy in protecting sheep against challenge with a genetically distinct virus, Ken06. The challenge strain in the current study has been shown to be highly virulent, causing typical RVF clinical symptoms in infected animals, including death^37^.

The recombinant subunit vaccine conferred complete protection from clinical disease by preventing pyrexia and viremia (as demonstrated by virus isolation), following virulent RVFV challenge in all vaccinated sheep (Fig. 3B). Although a low level of residual viral RNA was detected in one of the five vaccinated sheep at 3 dpc with borderline cycle threshold (Ct) cutoff values, no infectious virus particles were isolated from this animal by virus isolation/plaque assay. In marked contrast, mock-vaccinated animals rapidly developed viremia, pyrexia and two of the five animals died of acute RVF. These results suggest that the Gn/Gc subunit vaccine might provide sterilizing immunity against virulent RVFV challenge. This finding is significant, since to date, only a couple of reports have been made about RVFV sterilizing immunity in a ruminant model, using a subunit vaccine^28^,^32^. Recent studies reported that vaccines based on live attenuated RVFV can provide sterilizing immunity in sheep^13^,^39^. However, studies using these live vaccines^13^,^39^ as well as the subunit adenovirus-vectored vaccine^32^ did not detect viral titers, assessed by virus isolation or plaque assay, in the blood of infected animals, making it difficult to objectively qualify this claim.

Analysis of serum AST and BUN concentrations showed that all mock-vaccinated animals developed significantly elevated levels of these biochemical indicators (*P* = 0.0167 and *P* = 0.02, respectively) within the first three days following virulent challenge (Fig. 2AB). Increased serum levels of AST following virulent RVFV infection are generally indicative of impairment of hepatocellular function, whereas elevated BUN concentrations are associated with renal functional impairment^24^,^37^,^42^. In stark contrast, following virulent challenge, vaccinated animals maintained their serum AST and BUN concentrations within normal physiological ranges until the study’s endpoint (Fig. 2A,B). Pathology results support these vaccine efficacy data and show protection of the sheep from liver and kidney disease. In contrast to unvaccinated sheep, no gross lesions were seen in vaccinated sheep at necropsy and no RVFV-attributable histopathologic lesions were detected in examined tissues. Although individual vaccinated sheep did have some common background lesions, such as a mild lymphoplasmacytic renal pelvic inflammation and low numbers of hepatic peri-portal lymphocytes and plasma cells, these lesions were morphologically inconsistent with RVF and negative for viral antigen by IHC. Taken together, these results suggest that the recombinant subunit vaccine provided complete protection from RVF-associated biochemical alterations and pathology.

Monitoring of host immune responses to vaccination indicated that all vaccinated animals seroconverted by 14 dpv (Fig. 4A) and exhibited time-dependent increase in antibody activity (Fig. 4A–F). Similarly, neutralizing antibody titers, the established correlate of RVFV protection^35^,^43^, were detectable in all vaccinated animals within two weeks at 14 dpv (Table 1). Administration of the second vaccine dose at 21 dpv resulted in an anamnestic response in all vaccinated animals (Fig. 4A; Table 1). Interestingly, following virulent challenge, none of the vaccinated animals demonstrated challenge-induced anamnestic immune responses, which suggests that the neutralizing antibody titers were effective in preventing and suppressing viral replication. In contrast, the three surviving mock-vaccinated animals developed detectable neutralizing titers at 7 dpc. In this study, animals were subjected to virulent challenge at 14 days post-booster, which is rather short. Thus, future studies should assess challenge at least 28 days post-booster.

In summary, the immunity induced by our recombinant subunit vaccine indicated that the vaccine was highly efficacious, and given the safety profile of subunit vaccines, suitable for control of RVF in non-endemic as well as endemic areas. The rectal temperature profiles, and the virological and serological assays demonstrated that a robust and sterilizing immune response was generated in the vaccinated animals challenged with Ken06 RVFV strain. Secondly, the vaccine meets a key attribute for use as an eradication tool, i.e. DIVA compatibility, when used in combination with RVFV recombinant N antibody ELISA^19^,^44^,^45^. Significantly, our vaccine constructs, while based on the sequence of the prototype ZH548 strain^12^, provided protection against a genetically heterologous virus strain (Ken06). Considering the lack of serological diversity among different strains of RVFV^46^, it is plausible that the recombinant subunit vaccine could provide broad protection against genetically diverse strains of RVFV. Given the global increase in international trade and travel, production and stockpiling of a vaccine with a high safety profile and efficacy against challenge with a genetically distinct virus, represent an attractive strategy to ensure better preparedness for future introductions (accidental or intentional) and disease outbreaks.

Finally, in the near future, it will be important to evaluate a single-dose regimen for the vaccine including the use of different improved adjuvants designed to enhance vaccine immunogenicity and host immune responses. Such improved adjuvants should induce early-onset immune response and higher amount of neutralizing antibodies, mainly by polarizing the immune response towards antibody secreting B cells, in a single vaccination regimen. Remarkably, the recombinant subunit vaccine after single vaccination induced mean neutralizing antibody titers of 38 (range = 10–80) and 64 (range = 40–80) at 14 and 21 dpv, respectively (Table 1). These titers are within the range associated with protection in other livestock challenge studies^13^,^14^, suggesting possible administration of the subunit vaccine as a single-dose vaccine regimen. Importantly, in a previous study, the recombinant Gn/Gc subunit vaccine induced similar mean neutralizing antibody titers of 62 and 77, at 14 and 21 dpv, respectively, in sheep after single vaccination^19^. Following second vaccination, neutralizing antibody titers persisted above the protective threshold^10^ in all vaccinated animals for nearly a year post vaccination^19^. Furthermore, in most RVF endemic countries, vaccination is recommended prior to the summer rainfall season when vector abundance and activity increases and the risk of possible disease outbreak is anticipated^14^. It is therefore logical to recommend that a single dose vaccine regimen should induce protective titers lasting minimum 6 months to be effective in preventing possible disease outbreaks. Further evaluation of the efficacy of the subunit vaccine in other ruminant livestock species and non-human primates, as well as performing field testing of the vaccine in RVF endemic regions, notably in sub-Saharan Africa, are also the initial next steps towards developing a safe and efficacious vaccine for use in both livestock and humans in RVF endemic and non-endemic regions.

## Methods

### Viruses and cells

The RVFV Kenya 2006-128b-15 (Ken06)^47^ isolate was provided by R. Bowen, Colorado State University through B. Miller, Centers for Disease Control, Fort Collins, CO. The Ken06 virus strain was propagated in a C6/36 *Aedes albopictus* cell line (ATCC, Manassas, VA) with MEM culture medium (Life Technologies, Grand Island, NY) supplemented with 10% fetal bovine serum (FBS; Sigma-Aldrich, St. Louis, MO) and 1x Penicillin/Streptomycin/Fungizone (PSF; Gibco, USA). The *A. albopictus* cell line was maintained at 28 °C, whereas virus-infected cells were maintained at 37 °C. MP12 is a non-virulent strain of RVFV, attenuated via chemical mutagenesis^12^ and was used as the viral stock in plaque reduction neutralization assays^19^,^37^. Vero MARU (Middle America Research Unit, Panama) cells were used for virus isolation and titration. The cells were grown in Medium M-199 (M199E) culture medium (Sigma-Aldrich) supplemented with 10% FBS and 1x PSF, and maintained in a 37 °C, 5% CO_2_ incubator.

### Recombinant baculovirus expression and purification of RVFV Gn and Gc glycoproteins

The cloning and creation of recombinant baculovirus constructs for expression of RVFV glycoproteins, Gn and Gc, has been described previously^19^,^48^. The ectodomain of Gn glycoprotein (Gne) was expressed, which hereafter will be referred to as Gn. Gc glycoprotein was expressed as a full-length protein. Recombinant protein expression was carried out using passage 2 (P2) or higher-passage recombinant baculovirus stocks (>10^7^ pfu/ml). The proteins were expressed with a carboxy-terminal 6xHistag, and purification using Ni-NTA Superflow resin (QIAGEN Inc., Valencia, CA) was performed as described previously^18^. Concentration of the purified proteins was measured by method of bicinchoninic acid (BCA) assay (Thermo Scientific, Rockford, IL) at an absorbance of 562 nm, using bovine serum albumin (Sigma-Aldrich, St. Louis, MO) as the protein standard. Aliquots of the protein were stored at −80 °C until used.

### Vaccine preparation

To prepare the subunit vaccine, recombinant Gn and Gc glycoprotein were formulated in montanide ISA25 VG (Seppic, France) to obtain an equal amount of 50 μg of each immunogen (Gn and Gc) per vaccine dose according to the manufacturer’s instruction.

### Animals, vaccination and viral challenge

Ten naïve healthy sheep (*Dorper x Katahdin cross*), aged 4–5 months, were obtained from a private breeder in Kansas, USA. The sheep were acclimated for seven days at the Large Animal Research Center (LARC; Kansas State University) and subjected to prophylactic treatment and deworming using Draxxin and Albendazole, respectively. All experimental protocols were performed blinded. The animals were divided into two groups (*n* = 5 per group). Group 1 was inoculated subcutaneously with 2 ml of the subunit vaccine composed of 50 μg of each of the glycoproteins Gn and Gc. Animals in group 2 served as mock-vaccinated controls and were inoculated with an equivalent volume of adjuvant only. Prevaccination blood samples were collected from all animals at 0 days post primary vaccination (dpv), and thereafter bled weekly at 7 to 35 dpv. The animals were monitored during the first three dpv for changes in rectal temperature and localized inflammation at the site of vaccine administration. Additionally, vaccination sites were monitored during a 35-day post-vaccination period for occurrence of erythema, tissue nodules or abscess formation. A second vaccination regimen (the booster) was administered at 21 dpv. At 28 dpv, the animals were relocated to a BSL-3Ag facility at the Kansas State University Biosecurity Research Institute (BRI). To assess the protective efficacy of the vaccine, at 35 dpv, corresponding to 0 days post challenge (dpc), all animals were challenged subcutaneously with 2 ml of 1 × 10^6^ PFU of Ken06 RVFV strain. Post-challenge, all animals were monitored daily for viremia including clinical signs rectal temperature changes. Blood samples for virological, immunological and blood chemistry analyses were collected daily from 0 to 7 days post-challenge (dpc). Post euthanasia on dpc 4 and 7, necropsies were performed and tissue samples were collected for histopathology. Research was performed under an Institutional Animal Care and Use Committee-approved protocol of Kansas State University in compliance with the Animal Welfare Act and other regulations relating to animals and experiments involving animals. All experimental protocols and procedures were approved by the Kansas State University Institutional Biosafety Committee (IBC, Registration #: 1004) and the Institutional Animal Care and Use Committee (IACUC, Protocol #: 3518).

### Viral RNA extraction and real-time PCR

Total RNA was extracted from serum using TRIzol-LS reagent (Life Technologies, Grand Island, NY) and the magnetic-bead capture MagMAX-96 total RNA Isolation kit (Life Technologies) as described previously^37^. Briefly, 100 μl of aqueous phase was added to 90 μl of isopropanol and 10 μl bead mix. Total sample RNA was washed four times with wash buffer (150 μl), then eluted in 30 μl of elution buffer. A published quadruplex real-time reverse transcriptase-polymerase chain reaction (RT-PCR) assay was used to detect each of the three RVFV RNA genome segments^47^. The cut-off cycle threshold (Ct) value was set at 35.

### Virus titration

Virus challenge material and sheep sera were titrated by standard plaque assay on Vero MARU cells. Briefly, confluent cell monolayers were inoculated with ten-fold serially diluted samples in M199E and incubated for 1h. Following adsorption, the inocula were replaced with a 1:1 mixture of 2% carboxymethyl cellulose (Sigma-Aldrich, St. Louis, MO) in 2x M199E (20% FBS and 2x PSF) and returned to the incubator. After 5 days, cells were fixed and stained with crystal violet fixative (25% formaldehyde, 10% ethanol, 5% acetic acid, 1% crystal violet). Virus titers were calculated to determine the PFU/ml.

### Serology

#### Immunogen-specific Indirect ELISA

Vaccine-induced seroconversion was monitored at 0 to 35 dpv. For this, anti-RVFV Gn-specific IgG antibodies were detected using the indirect enzyme linked immunosorbent assay (ELISA) method described previously^19^,^49^. Additionally, endpoint titers of Gn-specific antibodies in ten-fold serial dilutions were determined for each time point postvaccination using the indirect ELISA. The cut-off point for seroconversion was determined for each individual animal and was deduced by the addition of three standard deviations to the corresponding mean OD value of the pre-vaccination serum. Mean OD values equal to or greater than the cut-off value were considered positive seroconversion.

#### Plaque reduction neutralization assay

Assessment of anti-RVFV neutralizing antibody responses to vaccination was performed using the plaque reduction neutralization test (PRNT_80_) as described previously^19^. Briefly, aliquots of serum from each vaccinated sheep were diluted as follows: 1:10, 1:20, 1:40, 1:80, 1:160, 1:320, 1:640, and 1:1280 in 1x MEM containing 2% bovine serum albumin and 1% penicillin streptomycin. The stock of MP12 RVFV was diluted to 50 PFU in 250 μl of 1x MEM containing 4% bovine serum albumin (Sigma-Aldrich). Diluted serum (250 μl) was mixed with an equal volume of diluted MP12 virus and incubated at 37 °C for 1 h. Each mixture of serum plus RVFV was used to infect confluent monolayers of Vero E6 cells in 12-well plates. After 1 h adsorption at 37 °C and 5% CO_2_, the mixture was removed, and 1.5 ml of nutrient agarose overlay (1x MEM, 4% bovine serum albumin, and 0.9% SeaPlaque agar) was added to the monolayers. After 4–5 days incubation, the cells were fixed with 10% neutral buffered formalin for 3 h prior to removal of the agarose overlay. The monolayer was stained with 0.5% crystal violet in PBS, and plaques were enumerated. The calculated 80% titers (PRNT_80_) corresponded to the reciprocal titer of the highest serum dilution, which reduced the number of plaques by 80% or more relative to the virus control^19^.

### Blood chemistry

In our previous study, we reported that aspartate aminotransferase (AST) and blood urea nitrogen (BUN) are two key blood chemistry values most affected by virulent RVFV infection^37^. Thus in this study, analysis of serum concentrations of AST and BUN was performed using a VetScan VS2 Chemical Analyzer (Abaxis, Union City, CA) as described previously^37^.

### Pathology

Nine sheep, four vaccinated (the fifth vaccinated sheep was sacrificed due to injury) and five mock-vaccinated animals, were necropsied. Samples from the following tissues were collected at necropsy and placed in 10% neutral buffered formalin for at least 7 days: liver, spleen, left kidney, left adrenal gland (except from 4 dpc sheep), mesenteric (jejunal) lymph node, lung and eye. These samples were trimmed, placed in cassettes, dehydrated and embedded in paraffin. 4-μm sections were cut and placed on positively charged slides for histochemical staining and immunohistochemistry (IHC). Hematoxylin-and-eosin (H&E) stained tissues and IHC results were examined and scored where appropriate by a veterinary pathologist in a blinded fashion. Liver was scored on a scale from 0–3 as described previously^37^.

Immunohistochemistry for RVFV antigen was conducted as described previously^37^. Briefly, we used an auto-stainer based polymer detection type technique to screen tissues and then an avidin-biotin complex detection technique by hand on a representative subset of tissue sections, in order to take images with less background for this manuscript. Both protocols used the same primary antibody, a polyclonal rabbit anti-RVFV nucleocapsid protein antibody^50^. IHC was conducted on all liver, spleen, kidney, lung and adrenal samples. For lymph nodes, in order to clearly distinguish RVFV antigen IHC signal from hemosiderin and other brown pigments present inside macrophage cytoplasm, we developed an additional RVFV IHC using the same primary antibody and a different chromogen and counterstain. Briefly, slides were deparaffinized, rehydrated, antigen retrieved using a vegetable steamer technique in pH 6.0 citrate buffer with detergents (DAKO; Carpinteria, CA) for 20 min, blocked with 3% hydrogen peroxide for 10 min, serum blocked as per kit (VECTASTAIN Elite Kit (Rabbit IgG), Vector Labs; Burlingame, CA), incubated overnight at 4 °C with 1:500 dilution in TBS 1x of primary antibody, secondary antibody and ABC reagent applied as per kit, VECTOR VIP Peroxidase Substrate and VECTOR Methyl Green counterstain applied as per vendor instructions (Vector Labs) and mounted in Permount (Electron Microscopy Systems; Hatfield, PA). Image capture and post-processing of histopathology was conducted as described prior^37^.

### Statistical analysis

Analyses for statistically significant differences between vaccinated and control animals were performed for temperature, AST and BUN response values. Differences in mean AST and BUN response values between vaccinated and mock-vaccinated groups were analyzed using two-way ANOVA. Group mean values of temperature responses for vaccinated and mock-vaccinated groups per time point were derived and then analyzed using two-way ANOVA.

## Additional Information

**How to cite this article**: Faburay, B. *et al*. A Recombinant Rift Valley Fever Virus Glycoprotein Subunit Vaccine Confers Full Protection against Rift Valley Fever Challenge in Sheep. *Sci. Rep*. **6**, 27719; doi: 10.1038/srep27719 (2016).

## Figures and Tables

**Table 1:** Neutralizing antibody titers in sheep following vaccination and challenge assessed by plaque reduction neutralization assay (PRNT_80_).

Sheep ID	Group	PRNT_80_ Postvaccination titer					PRNT_80_ Postchallenge titer	
		0 dpv	7 dpv	14 dpv	21 dpv	28 dpv	0 dpc	7 dpc
62	Vac	–	–	80	40	1280	>1280	1280
64*	Vac	–	20	40	80	>1280	N/A	N/A
66	Vac	–	20	40	80	1280	1280	1280
67	Vac	–	–	10	80	1280	>1280	1280
70	Vac	–	–	20	40	1280	1280	1280
Mean		N/A	20^a^	38	64	1280	1280	1280
63	Mock	–	–	–	–	–	–	N/A
65	Mock	–	–	–	–	–	–	80
68	Mock	–	–	–	–	–	–	160
69	Mock	–	–	–	–	–	–	N/A
71	Mock	–	–	–	–	–	–	80
Mean		N/A	N/A	N/A	N/A	N/A	N/A	133^b^

Key: dpv = days postvaccination; dpc = days postchallenge; ^a^n = 2; ^b^n = 3; N/A = not applicable; *sheep #64 was sacrificed due to leg injury; (–) = no titer.

**Table 2:** Histopathology and immunochemistry for RVFV antigen.

Sheep ID	Group	DPC	Avg H Score		IHC	H Other Organs	IHC+
63	Mock	4	3		+	s, k, ln, lu	s, k, ln, lu
69	Mock	4	3		+	s, k, ln, lu	s, k, ln, lu
65	Mock	7	2		+	k	−
68	Mock	7	2		+	−	−
71	Mock	7	2		+	a	a
62	Vac	7	0		−	I	−
66	Vac	7	0		−	k	−
67	Vac	7	0		−	k	−
70	Vac	7	0		−	k	−

Avg H Score is average hepatic histopathology score on a scale from 0, no lesions to 3 severe lesions. DPC is days post challenge. IHC is the Liver RVFV IHC for viral antigen result. H Other Organs is histopathology in organs other than the liver. IHC + lists organs that were positive for viral antigen. Key: + = positive for viral antigen by IHC, − = negative for viral antigen on IHC, s = spleen, k = kidney, ln = lymph node, lu = lung, a = adrenal and i = intestine.
